# Validation of the Chinese Version of the Questionnaire for Impulsive-Compulsive Disorders in Parkinson's Disease

**DOI:** 10.3389/fneur.2021.731552

**Published:** 2021-12-07

**Authors:** Tian Xu, Lanxiao Cao, Wenying Long, Guohua Zhao

**Affiliations:** ^1^Department of Neurology, The Fourth Affiliated Hospital, Zhejiang University School of Medicine, Yiwu, China; ^2^Central Laboratory, The Fourth Affiliated Hospital, Zhejiang University School of Medicine, Yiwu, China; ^3^Department of Neurology, The Second Affiliated Hospital, Zhejiang University School of Medicine, Hangzhou, China

**Keywords:** Parkinson's disease, impulsive-compulsive disorders, impulse control and related disorders, validity, questionnaire

## Abstract

**Background:** Impulse control and related disorders (ICRDs) have gained recognition as a severe complication of Parkinson's disease (PD) and are connected to poor quality of life and devastating financial and social problems. This study aimed to evaluate the usefulness of the Questionnaire for Impulsive-Compulsive Disorders in Parkinson's Disease (QUIP) and estimate the risk factors for ICRDs in Chinese patients with PD.

**Methods:** 207 PD patients were assessed using the QUIP and evaluated for PD motor and nonmotor symptoms. ICRDs were diagnosed *via* interviews of patients or their caregivers, and the clinical characteristics of patients with and without ICRDs were compared.

**Results:** The sensitivity, specificity, positive predictive value, negative predictive value, and accuracy of the C-QUIP were 95.0, 83.4, 38.0, 99.4, and 84.5%. The prevalence of each disorder among participants diagnosed *via* interview was pathological gambling (0.5%), hypersexuality (1.9%), compulsive shopping (1.0%), binge eating (3.9%), hobbyism (1.9%), punding (0.5%), walkabout (0.5%), and dopamine dysregulation syndrome (2.9%). PD patients with ICRDs had longer PD duration, higher Hoehn and Yahr stage, Non-Motor Symptoms Scale (NMSS), and Hamilton-Depression Rating Scale (HAMD). Also, they received a larger total daily levodopa equivalent dose (LED), levodopa dosage, and dopamine agonist only LED (DA-LED) than did PD patients without ICRDs.

**Conclusions:** Given its psychometric properties, the C-QUIP is a valid and rapid screening instrument for assessing of ICRDs in PD patients. Higher Hoehn and Yahr staging, NMSS and HAMD scores, a larger mean LED and levodopa dosage are risk factors for ICRDs.

## Introduction

In addition to its characteristic motor signs and symptoms, Parkinson's disease (PD), a common degenerative neurological disorder, has many non-motor signs and symptoms, olfactory dysfunction, constipation, depression, apathy, rapid-eye-movement sleep behavior disorder, and sleep disturbances. Impulse control disorders (ICDs) involve repetitive, excessive, and compulsive behaviors driven by intense desire (1). Patients often find it difficult to control themselves even though these behaviors cause harm to themselves or others. ICDs include pathological gambling, hypersexuality, compulsive shopping, and binge eating. As the presence of ICDs in PD has received increasing attention, its clinical symptom spectrum has expanded to include impulse control and related disorders (ICRDs), specifically, dopamine dysregulation syndrome (DDS), hobbyism, and punding. These behaviors are linked by their repetitive nature based on incentives or rewards (2). It is crucial to identify ICRDs because these behaviors can lead to serious financial problems and impair the quality of life of both patients and their caregivers. Early interventions, i.e., medication adjustment, can effectively improve symptoms and prevent severe consequences (3).

To standardize the clinical diagnosis and study of ICRDs in PD, the International Parkinson and Movement Disorder Society recommends using the Questionnaire for Impulsive-Compulsive Disorders in Parkinson's Disease (QUIP) and Questionnaire for Impulsive-Compulsive Disorders in Parkinson's Disease Rating Scale (QUIP-RS) for assessments of ICRDs in patients with PD (4). The QUIP is a self-report tool for screening ICRDs (4) but requires a follow-up interview to confirm the diagnosis. The validation of the QUIP has been tested in many languages (5–7) but not in Chinese. Therefore, our study aimed to evaluate the QUIP's usefulness and estimate the risk factors for ICRDs in Chinese patients with PD.

## Materials and Methods

### Participants

We recruited 207 consecutive patients diagnosed with idiopathic PD in the Department of Neurology at the Fourth Affiliated Hospital Zhejiang University School of Medicine and the Second Affiliated Hospital, Zhejiang University School of Medicine, between October 2018 and January 2021. The clinical diagnosis of PD was based on criteria from the Parkinson's Disease Society Brain Bank in London (8). Patients with secondary Parkinsonism or Parkinson-plus syndrome were excluded from the study. All participants provided written informed consent and the Ethics Committee of The Fourth Affiliated Hospital Zhejiang University School of Medicine approved the study.

### Assessments

Participants' demographic and clinical characteristics (gender, age, age at onset of PD, duration of PD, and education) were evaluated. Patients' medications and dosages for PD were recorded at the time of the assessment; the total daily levodopa equivalent dose (total daily LED) (9), levodopa equivalent dose (LED), and dopamine agonist only LED (DA-LED) were calculated. Clinical assessments were performed using the following measures: Parts I, II, and III of the Unified Parkinson's Disease Rating Scale (UPDRS) (10), dyskinesia, the Hoehn and Yahr staging scale (11), the Mini-Mental State Examination (MMSE) (12), the Chinese version of the 39-item Parkinson's Disease Questionnaire (C-PDQ39) (13), the Non-Motor Symptoms Scale (NMSS), the Hamilton-Anxiety Rating Scale (HAMA), and the Hamilton-Depression Rating Scale (HAMD).

**Figure 1:**
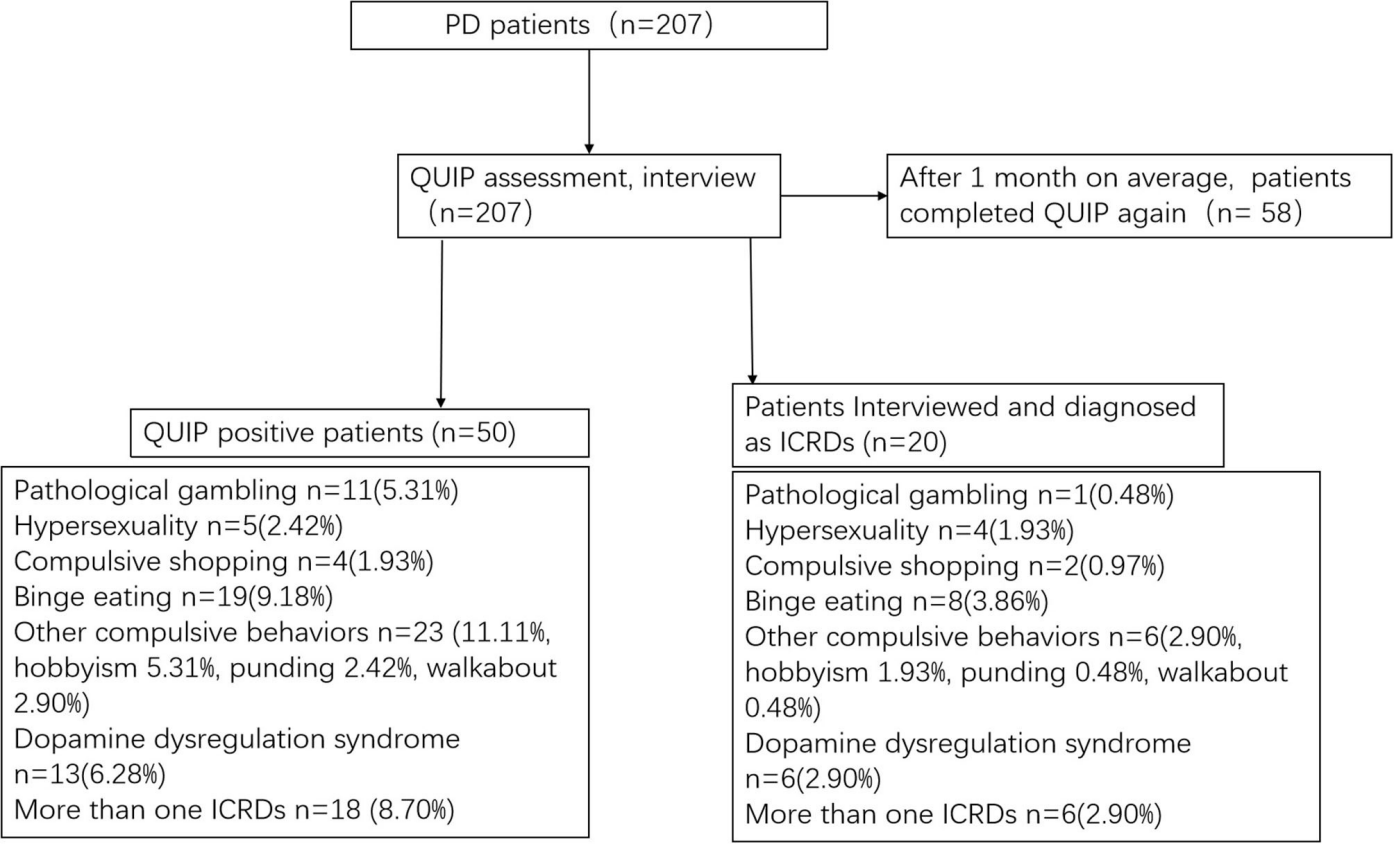
Diagnostic flow chart for this study.

The original QUIP includes a full and short version, and both assess ICRDs currently or anytime during PD (QUIP-Current-Full, QUIP-Anytime During PD-Full, QUIP-Current-Short, QUIP-Anytime During PD-Short) (6). The QUIP contains 30 questions and its shortened version (QUIP-S) contains 13 questions, and the specific questions and overall structure of QUIP-S were not modified. The reason for choosing the time frame of “anytime during PD” is that plenty of PD patients experienced ICRDs during PD and are currently asymptomatic due to clinical management, but may be at increased risk of developing ICRDs in the future. As no differences were found in the sensitivity between these versions (6), they were all recommended for routine use, and we used only the QUIP-Anytime During PD-Short. All patients were interviewed by an experienced movement disorder neurologist, who was blind to patients' QUIP results and their final diagnoses. We diagnosed pathological gambling, hypersexuality, compulsive shopping, binge eating, hobbyism, punding, and DDS using the various diagnostic criteria listed in a review by Voon and Fox (2). We constructed the C-QUIP using forward and backward translations of the original QUIP, and the primary developer of the original questionnaire (DW) proofread the final version. We also obtained permission from the developer to translate the QUIP to Chinese. The specific questions and structure of C-QUIP are the same as the Short-Anytime During PD version of the original QUIP (6). The patient is screened as positive for each ICD if ≥1 affirmative answer to any question (The optimal cutoff point). The test-retest reliability of the C-QUIP was evaluated by performing assessments using the questionnaire a second time within 1 month after the first assessment. Figure 1 demonstrates the diagnostic flow chart for this study.

### Statistical Analysis

Data were analyzed using SPSS for Windows version 19 (IBM Corp, Armonk, NY). The results are presented as mean ± standard de*via*tion. The sensitivity, specificity, positive predictive value (PPV), negative predictive value (NPV), and accuracy of the QUIP for the detection of symptoms were calculated. Between-group differences (positive vs. negative diagnosed as ICRDs) were analyzed using the Wilcoxon-Mann-Whitney test for continuous variables and the χ^2^ test or Fisher's Exact test for categorical variables. *P* < 0.05 was considered statistically significant. The Guttman split-half coefficient was used to evaluate the test-retest reliability of the C-QUIP.

## Results

This study analyzed data from 207 patients (63.8% males) aged 60.8 ± 8.7 years with a PD duration of 4.1 ± 3.7 years. Half of the patients or their caregivers take <2 min to complete the questionnaire. Among the 207 patients, 24.2% screened positive on the C-QUIP, but only 9.7% were diagnosed eventually with ICRDs. Their behaviors and medications are presented in Table 1. Binge eating was the most common symptom with a frequency of 3.9%, followed by DDS (2.9%), hypersexuality (1.9%), and hobbyism (1.9%). Approximately 5.3% of the patients screened positive for compulsive gambling, but only one person met the diagnostic criteria, and 2.9% had more than one type of ICRDs (Table 1).

**Table 1:** Demographic and clinical characteristics of 20 PD-ICRDs patients.

**Case**	**Gender**	**Age (year)**	**AAO (year)**	**Medication**	**Dyskinesia**	**Total LED(mg)**	**ICRDs**
1	Male	64	60	Levodopa	No	375	Eat^a^
2	Female	68	62	Levodopa, Pramipexole, Amantadine, Selegiline	No	1,150	DDS^c^
3	Male	57	53	Levodopa, Pramipexole, Amantadine	No	750	Eat, Sex^b^
4	Male	41	37	Levodopa, Sinemet, Entacapone, Piribedil, Selegiline, Amantadine	Yes	1092.75	DDS
5	Male	60	58	Levodopa, Piribedil, Selegiline	No	425	DDS
6	Male	68	61	Levodopa, Pramipexole, Selegiline	No	525	Eat
7	Male	51	50	None	No	0	Walk about
8	Male	68	66	Levodopa	No	400	Eat
9	Male	41	36	Levodopa, Pramipexole	Yes	1,350	Sex, Punding
10	Female	61	51	Levodopa, Sinemet, Entacapone, Pramipexole, Selegiline	No	1,688	Hobbyism, DDS
11	Male	49	44	Levodopa, Selegiline	No	300	Eat
12	Female	66	60	Levodopa	No	500	Buy^d^, Eat
13	Male	58	55	Levodopa, Pramipexole	No	262.5	Hobbyism
14	Male	55	47	Levodopa, Pramipexole, Amantadine	No	750	Sex, Eat, Hobbyism, DDS
15	Male	69	59	Levodopa, Pramipexole, Comtan	No	798.75	Punding, DDS
16	Male	53	46	Levodopa, Pramipexole, Amantadine	No	1447.5	Gamble^e^
17	Male	71	65	Levodopa, Pramipexole, Selegiline	No	612.5	Sex
18	Male	49	44	Levodopa, Pramipexole	No	725	Hobbyism
19	Male	61	59	Levodopa, Pramipexole	No	0	Eat
20	Female	53	51	none	No	0	Buy

a *Eat, binge eating.*

b *Sex, hypersexuality.*

c *DDS, dopamine dysregulation syndrome.*

d *Buy, compulsive shopping.*

e *Gamble, pathological gambling.*

*PD, Parkinson's disease; ICRDs, Impulse control and related disorders; AAO, Age at onset; LED, Levodopa equivalent dose; IBs, Impulsive and compulsive behaviors*.

The sensitivity, specificity, positive predictive value (PPV), negative predictive value (NPV), and accuracy of the C-QUIP to detect each of the behaviors are presented in Table 2. We also compared C-QUIP with the original version and Japanese version of QUIP. The sensitivity and specificity for most of the measures on the C-QUIP were above 90%, and the NPV for each impulsive and compulsive behavior was close to 100%. The sensitivity of the C-QUIP for the detection of hypersexuality was 80% because one patient concealed this symptom, and it was not reported until a follow-up interview with his wife. The test-retest reliability assessment was performed with 58 patients, and the Guttman split-half coefficient for the total C-QUIP was 0.869.

**Table 2:** The sensitivity, specificity, positive predictive value, negative predictive value of C-QUIP.

		**C-QUIP**	**Gamble^a^**	**Sex^b^**	**Buy^c^**	**Eat^d^**	**Other repetitive and compulsive behaviors**	**DDS**
This study	Sensitivity	95.0%	100%	88.0%	100%	100%	100%	100%
	Specificity	83.4%	95.2%	99.5%	99.0%	94.5%	91.5%	96.5%
	PPV	38.0%	9.1%	80.0%	50.0%	42.1%	26.1%	46.2%
	NPV	99.4%	100%	99.5%	100%	100%	100%	100%
	Accuracy	84.5%	95.2%	99.0%	99.0%	94.7%	91.8%	96.6%
Weintraub et al. Study 2009 (6)	Sensitivity	-	91%	100%	80%	86%	-	-
	Specificity	-	95%	90%	91%	85%	-	-
	PPV	-	59%	48%	38%	21%	-	-
	NPV	-	99%	100%	99%	99%	-	-
Tanaka et al. Study 2013 (5)	Sensitivity	-	83.3%	100%	100%	100%	-	100%
	Specificity	-	90.6%	100%	92.0%	92.0%	-	81.7%
	PPV	-	38.5%	100%	30.0%	30.0%	-	11.8%
	NPV	-	98.7%	100%	100%	100%	-	100%

a *Gamble, pathological gambling.*

b *Sex, hype-sexuality.*

c *Buy, compulsive shopping.*

d *Eat, binge eating.*

*C-QUIP, Chinese version of the Questionnaire for Impulsive-Compulsive Disorders in Parkinson's Disease; DDS, dopamine dysregulation syndrome*.

The clinical characteristics of PD patients with ICRDs (PD-ICRDs) and PD patients without ICRDs (PD-nonICRDs) are shown in Table 3. The mean scores on Parts I, II, and III of the UPDRS were 2.0, 9.4, and 23.4, respectively. Thirteen patients (6.3%) had dyskinesia; the mean Hoehn and Yahr stage was 2.0; the mean PDQ39 score was 17.1; the mean NMSS score was 22.0; the mean MMSE score was 25.4; and the mean HAMD and HAMA scores were 6.9 and 9.4, respectively. Levodopa usage was reported in 79.2% of the patients (LED 283.4 ± 220.7 mg/d), DA was used by 49.3% of them (DA LED 45.6 mg/d), and the total daily LED was 410.9 mg/d.

**Table 3:** Comparisons of demographic factors, clinical symptoms, and medications between patients with and without ICRDs.

	**Overall**	**ICRDs**	**Non-ICRDs**	***P***
**Demographic factors**	*N* = 207	*N* = 20	*N* = 187	
Male: female	132:75	15:5	117:70	0.272
Age	60.8 ± 8.7	58.3 ± 2.0	61.1 ± 0.6	0.182
Age at onset	57.1 ± 8.8	53.6 ± 1.9	57.4 ± 0.6	0.071
Duration	4.1 ± 3.7	4.8 ± 0.6	4.0 ± 0.3	0.040^*^
Education years	8.2 ± 4.0	9.6 ± 1.0	8.0 ± 0.3	0.316
**Clinical symptoms**				
Hoehn-Yahr scale	2.0 ± 1.1	2.3 ± 0.1	2.0 ± 0.1	0.018^*^
UPDRS-I	2.0 ± 2.1	2.1 ± 0.5	2.0 ± 0.2	0.527
UPDRS-II	9.4 ± 5.5	11.5 ± 1.3	9.2 ± 0.4	0.094
UPDRS-III	23.4 ± 12.4	22.9 ± 2.4	23.5 ± 0.9	0.976
NMSS	22.0 ± 16.8	29.5 ± 3.6	21.2 ± 1.2	0.013^*^
PDQ-39	17.1 ± 16.4	28.3 ± 5.7	15.9 ± 1.1	0.060
MMSE	25.4 ± 3.2	26.4 ± 0.7	25.2 ± 0.2	0.103
HAMD	6.9 ± 4.9	9.4 ± 1.3	6.7 ± 0.3	0.039^*^
HAMA	9.4 ± 5.5	10.7 ± 1.6	9.2 ± 0.4	0.579
Sexual dysfunction (+: –)	72:135	7:13	65:122	0.983
Dyskinesia (+:–)	13:194	2:18	11:176	0.813
**Medication**				
Total LED(mg/d)	410.9 ± 318.2	679.5 ± 119.3	382.2 ± 20.0	0.011^*^
DA LED(mg/d)	45.6 ± 58.0	85.6 ± 19.3	41.3 ± 3.9	0.020^*^
L-dopa LED(mg/d)	283.4 ± 220.7	457.8 ± 78.7	264.7 ± 14.2	0.005^*^
Agonist use (+:–)	102:105	13:7	89:98	0.139
Levodopa use (+:–)	164:43	17:3	147:40	0.704
MAOI LED	22.5 ± 9.2	22.5 ± 9.2	18.9 ± 2.7	0.549
Amantadine LED	65.0 ± 26.4	65.0 ± 26.4	49.5 ± 6.7	0.735

*C-PDQ39, Chinese version of the 39-item Parkinson's Disease Questionnaire; HAMA, Hamilton Anxiety Rating Scale; HAMD, Hamilton-Depression Rating Scale; ICRDs, Impulse control and related disorders; LED, Levodopa equivalent dose; MAOI, monoamine oxidase inhibitor; MMSE, Mini-Mental State Examination; NMSS, Non-Motor Symptoms Scale; UPDRS, Unified Parkinson's Disease Rating Scale.*

* *P < 0.05*.

These patients were then divided into two groups based on whether they had been diagnosed with an ICRD to compare differences in their demographic characteristics, clinical symptoms, and medications. We found that PD-ICRDs had a longer duration of PD (*P* = 0.040), higher Hoehn and Yahr stage (*P* = 0.018), higher NMSS (*P* = 0.013) and HAMD (*P* = 0.039) scores; and a larger total LED (*P* = 0.011), levodopa dosage (*P* = 0.005) and DA LEDs (*P* = 0.020), compared with PD-nonICRDs. The mean scores on the UPDRS-II (*P* = 0.094) and the PDQ-39 (*P* = 0.060) of PD-ICRDs were higher than those of PD-nonICRDs, but their differences were not statistically significant.

## Discussion

The QUIP is a self-administered questionnaire to assess a comprehensive range of ICRDs in PD patients. Its usefulness has been verified in different countries, such as Japan and Germany (5–7). Several studies have examined ICDs or ICRDs in Chinese patients with PD (14–18), but they used different assessment instruments. Hence, the between-study results could not be compared. The QUIP was designed as a suitable screening tool rather than a diagnostic or rating tool for ICRDs in Parkinson's disease. A high NPV is crucial for a screening tool and this study's NPVs for most subtypes of ICRDs were 100%, with an accuracy >90%, which are similar detection rates as those of previous studies, indicating that the possibility of ICRDs would be extremely low in a patient with PD with a negative QUIP screening. As a brief, comprehensive and self-rated screening questionnaire, QUIP-S consumes only a very short time to complete without sacrificing diagnostic capability. There are several cons to note. First, it is necessary to confirm the diagnosis through a follow-up interview for the low PPVs and high false-positive rates. Concealment of one's symptoms, such as hypersexuality, whether intentional or not, should be considered, as it can be expected to be detected *via* a subsequent interview, especially one with the patient's spouse or caregivers. Second, it does not assess the severity of these behaviors, which is important for monitoring changes in these behaviors. Overall, the C-QUIP is practical as a questionnaire for rapid screening for ICRDs in clinical care and research.

Studies of different populations have reported prevalence rates of ICRDs in PD, ranging from 10.1 to 34.2% in countries outside of China (19, 20). The largest study to date has estimated that ICDs affect 13.6% of patients with PD (21), although the number of patients varies widely across samples. A multi-center longitudinal study found the 5-year cumulative incidence of ICDs was 46.1% (22). The percentage of positive responses for any of the ICRDs in our study was 9.15%, much lower than the rates in Western countries but similar to the rates reported in other studies conducted in China (15, 16). Another Chinese study identified ICDRs in 31.0% of patients with PD, but it used only the QUIP (without a follow-up interview) to clarify the diagnosis, which resulted in higher scores. The lower percentage of ICRDs in Chinese patients with PD than in Western countries may be attributable to lifestyle, cultural background, and medication differences. For instance, patients who self-reported pathological gambling in the QUIP did not meet the diagnostic criteria. Gambling is illegal in China; thus, the prevalence of pathological gambling is lower to some extent.

Previous studies have indicated that numerous controllable and uncontrollable factors are related to the development of ICRDs in PD. Uncontrollable risk factors include male gender (14, 18, 20, 23), young age (5, 20, 23, 24), early age at onset of the disease (5, 20, 23, 24), disease duration (5, 14, 20), and family history of impulsivity (21). Generally speaking, the positive rate of ICDRs in patients with PD is higher in males than in females (14, 18, 20, 23). A multi-center study that assessed Italian patients treated for PD for more than 2 years showed that 32.5% (223/686) of the males and 21.7% (83/383) of the females screened positive for ICDs (20). The gender difference was more pronounced in the analysis of specific ICRDs; hypersexuality was more common among male patients, while compulsive shopping and binge eating were more common among women. Controllable risk factors include smoking (20, 21), alcohol consumption (15, 20), depression (14, 20, 23, 25, 26), the use of DA (15, 21, 23, 25, 26) and levodopa (21), and the dosages of DA (14–17) and levodopa (5, 18). Chinese patients with PD are usually treated with lower dosages of medications. Zhang et al. found a DA dosage >1 mg/d was independently associated with ICRDs in PD (17). Dopamine agonists, such as pramipexole and ropinirole, have a higher affinity for D3 receptors than D1 or D2 receptors (27). D3 receptors are concentrated in the ventral striatum, ventral putamen, globus pallidus, and the medial dorsal nucleus of the thalamus, where they play a role in the mesolimbic reward pathways (28). We found an association of ICRDs with the total daily LED and levodopa dosage. The DA LED in PD-ICRDs was higher than in PD-nonICRDs, but the difference was not statistically significant. The total LED (410.9 ± 318.2 mg/d) and DA LED (45.6 ± 58.0 mg/d) in our study were much lower than those reported in studies conducted in countries outside of China.

Several previous studies found a close relationship between ICRDs and dyskinesia (14, 17). The ICARUS study found more than half of patients with PD and dyskinesia had ICRDs (20), and both ICRDs and dyskinesia were associated with dopaminergic drug therapy. Furthermore, they share common risk factors, including early age at onset of the disease, duration of the disease, and polymorphism of dopamine receptor genes, suggesting they might have similar pathogenic mechanisms (29). However, our study did not find a relationship between ICRDs and dyskinesia.

The limitations of our study include its cross-sectional design, lack of a longitudinal follow-up, and the small sample size of participants. As our study population consisted of only patients who could visit our hospital, we did not analyze data from a random sample. Besides, it is inadequate to complete multiple regression analysis to clarify the risk factors related to ICBs.

## Conclusions

In conclusion, we verified the usefulness of the C-QUIP as a rapid screening questionnaire for ICRDs, reported the prevalence and assessed possible risk factors for ICRDs in Chinese patients with PD. Case reports have suggested that reducing or discontinuing dopamine agonists often relieves symptoms of ICRDs (30). However, this medical adjustment might be difficult for some patients; thus, identifying ICRDs in patients with PD will help clinicians make therapeutic decisions and avoid severe consequences.

## Data Availability Statement

The raw data supporting the conclusions of this article will be made available by the authors, without undue reservation.

## Author Contributions

TX: data acquisition for the work and original draft preparation. LC: statistical analysis and interpretation of data for the work. WL: revising the work critically for important intellectual content. GZ: design of the work and revision. All authors contributed to the article and approved the submitted version.

## Funding

This work was supported by Research Foundation of Zhejiang Health (2020RC061) and the Jinhua Science and Technology Bureau Project (2020–3–004). The funders had no role in study design, data collection and analysis, decision to publish, or preparation of the manuscript.

## Conflict of Interest

The authors declare that the research was conducted in the absence of any commercial or financial relationships that could be construed as a potential conflict of interest.

## Publisher's Note

All claims expressed in this article are solely those of the authors and do not necessarily represent those of their affiliated organizations, or those of the publisher, the editors and the reviewers. Any product that may be evaluated in this article, or claim that may be made by its manufacturer, is not guaranteed or endorsed by the publisher.
